# Exploring the natural products chemical space to abrogate the F3L-dsRNA interface of monkeypox virus to enhance the immune responses using molecular screening and free energy calculations

**DOI:** 10.3389/fphar.2023.1328308

**Published:** 2024-01-10

**Authors:** Muhammad Suleman, Tanveer Ahmad, Khadim shah, Norah A. Albekairi, Abdulrahman Alshammari, Abbas Khan, Dong-Qing Wei, Hadi M. Yassine, Sergio Crovella

**Affiliations:** ^1^ Laboratory of Animal Research Center (LARC), Qatar University, Doha, Qatar; ^2^ Center for Biotechnology and Microbiology, University of Swat, Swat, Pakistan; ^3^ Department of Pharmacology and Toxicology, College of Pharmacy, King Saud University, Riyadh, Saudi Arabia; ^4^ Department of Bioinformatics and Biostatistics, School of Life Sciences and Biotechnology, Shanghai Jiao Tong University, Shanghai, China; ^5^ School of Medical and Life Sciences, Sunway University, Sunway City, Malaysia; ^6^ Biomedical Research Center, Qatar University, Doha, Qatar; ^7^ College of Health Sciences-QU Health, Qatar University, Doha, Qatar

**Keywords:** monkeypox, dsRNA, drug screening, MD simulation, binding free energy

## Abstract

Amid the ongoing monkeypox outbreak, there is an urgent need for the rapid development of effective therapeutic interventions capable of countering the immune evasion mechanisms employed by the monkeypox virus (MPXV). The evasion strategy involves the binding of the F3L protein to dsRNA, resulting in diminished interferon (IFN) production. Consequently, our current research focuses on utilizing virtual drug screening techniques to target the RNA binding domain of the F3L protein. Out of the 954 compounds within the South African natural compound database, only four demonstrated notable docking scores: −6.55, −6.47, −6.37, and −6.35 kcal/mol. The dissociation constant (KD) analysis revealed a stronger binding affinity of the top hits 1-4 (−5.34, −5.32, −5.29, and −5.36 kcal/mol) with the F3L in the MPXV. All-atom simulations of the top-ranked hits 1 to 4 consistently exhibited stable dynamics, suggesting their potential to interact effectively with interface residues. This was further substantiated through analyses of parameters such as radius of gyration (Rg), Root Mean Square Fluctuation, and hydrogen bonding. Cumulative assessments of binding free energy confirmed the top-performing candidates among all the compounds, with values of −35.90, −52.74, −28.17, and −32.11 kcal/mol for top hits 1-4, respectively. These results indicate that compounds top hit 1-4 could hold significant promise for advancing innovative drug therapies, suggesting their suitability for both *in vivo* and *in vitro* experiments.

## Introduction

Monkeypox, resulting from infection with the monkeypox virus (MPXV), a member of the orthopoxvirus genus, has traditionally been prevalent in central and western Africa (Durski et al., 2018; Doshi et al., 2019). However, since May 2022, occurrences of monkeypox in humans have surfaced in numerous countries outside its endemic regions. The global attention towards the disease has heightened due to an increasing number of confirmed cases and unusual instances of human-to-human and community transmission. The situation has reached over 79,000 confirmed cases spanning across 100+ countries, leading the World Health Organization (WHO) to declare it a Public Health Emergency of International Concern (PHEIC) on 23 July 2022 (Bunge et al., 2022; Guarner et al., 2022; Thornhill et al., 2022).

The monkeypox virus is characterized as a double-stranded DNA virus with a genome size measuring 197 kb. Its genetic makeup encompasses over 197 distinct nonoverlapping open reading frames (ORFs) (Manes et al., 2008; Iwasaki and Medzhitov, 2010; Bosworth et al., 2022; Khan et al., 2023). Within the virus’s structure, notable components include membrane proteins, structural proteins, and DNA-dependent RNA polymerase (Manes et al., 2008). The primary modes of transmission for the monkeypox virus involve the dispersion of large respiratory droplets, close personal contact with individuals exhibiting skin lesions, and the potential for contamination from objects they have come into contact with (Bosworth et al., 2022). The ongoing epidemic is having a more pronounced impact on individuals identifying as gay or bisexual, along with other men engaging in sexual activities with men, indicating a possible amplification of the infection’s spread within sexual networks. Clinical manifestations of monkeypox in humans closely resemble those observed in smallpox, featuring symptoms such as fever, rash, and skin lesions (Khan et al., 2023).

Cells of the innate immune system become active upon the attachment of pathogen-associated molecular patterns (PAMPs) using their PRRs (pattern-recognition receptors). Among the most prevalent PRRs are MDA5 (melanoma differentiation-associated protein 5), CLRs (C-type lectin-like receptors), RLR (RIG-1-like receptor), TLRs (toll-like receptors) and PKR (cytosolic protein kinase R) (Uematsu and Akira, 2006; Iwasaki and Medzhitov, 2010). When viral genetic material is detected, a response is triggered involving the collaboration of kinases such as TANK-Binding Kinase 1 (TBK1) and IкB Kinase Epsilon (IKK-ε). These kinases work together to add phosphate groups to IRF3, NF-kB (nuclear factor kappa B), and IRF7 (interferon regulatory factor 7) (Kawai and Akira, 2006). Typically, activation of RIG/MDA5 prompts the movement of IRF-3 into the cell nucleus, leading to subsequent processes.

Analysis of genomic data has uncovered striking similarities in the genetic sequences of MPXV and VACV. These two viruses share about 92% similarity at the nucleotide level and 88% similarity in terms of their protein sequences. Notably, the F3 protein in MPXV and the E3 protein in VACV exhibit homology, with strong structural and functional resemblances (Arndt et al., 2015). The C-terminal dsRNA-binding domain of the F3 protein remains intact and functional. However, a segment comprising the initial 37 amino acids of the Z-NA binding domain is absent (Yuwen et al., 1993; Langland and Jacobs, 2004). MPXV closely mimics the wild-type VACV in terms of its response to interferon (IFN) and its host range. It outperforms a VACV mutant with a corresponding N-terminal deletion in E3 by more efficiently dampening the cellular antiviral immune response. MPXV employs a strategy aimed at inhibiting type I IFN responses, effectively circumventing the host’s innate antiviral immunity. Studies indicate a noticeable increase in double-stranded RNA (dsRNA) during MPXV infection. Interestingly, the MPXV F3 protein possesses the capability to bind to dsRNA molecules, effectively sequestering them and preventing their interaction with recognized pathogen-sensing receptors like MDA-5, RIG-I, and PKR. Specifically, it disrupts the phosphorylation of PKR and eukaryotic translation initiation factor 2 (eIF2α) in the presence of dsRNA, resulting in a cessation of protein synthesis and a decrease in interferon (IFN) production. IFNs are crucial signaling proteins in the immune response. (Arndt et al., 2015; Arndt et al., 2016).

Currently, there are no targeted therapies available for monkeypox; however, certain approaches have shown effectiveness in addressing outbreaks. These include antiviral drugs, VIG (vaccinia immune globulin), and employment of the smallpox vaccine (Dryvax). Notably, the use of the Dryvax vaccine has limitations, as it can lead to unfavorable outcomes such as immune system irregularities in both the vaccinated person and those in close proximity (Haider et al., 2022). Throughout human history and the course of evolution, the utilization of natural products has been played a pivotal role in treating various diseases. Essential oils and extracts obtained from plants and animals are recognized as a significant reservoir of bioactive compounds (Donia and Hamann, 2003; Kumar et al., 2013; Lee and Hur, 2017; Ahmad et al., 2020; Denaro et al., 2020). The therapeutic properties of natural products have found extensive applications in fields like pharmaceuticals and ethnobotany, encompassing areas such as inflammation, cancer, oxidative stress, and viral infections. Many bioactive agents derived from natural sources have demonstrated effectiveness against a range of viruses including Dengue virus, Coronaviruses, Enterovirus, Hepatitis B, Influenza virus, and HIV (Lin et al., 2014; Hussain et al., 2017). Bioinformatics provides powerful computational tools such as virtual drug design which is a crucial strategy in the fight against infections as it offers a swift and effective method to pinpoint potential drug candidates. This approach specifically targets proteins implicated in the advancement of an infection, allowing scientists to streamline the selection of promising drug candidates before investing resources and time in laborious experimental procedures. However, the molecular dynamic simulation analysis offering detailed insights into the behavior of drug-protein complexes at the molecular level (Kumar et al., 2021a; Kumar et al., 2021b; Kumar and Doss, 2021; Tayubi et al., 2022; Abduljaleel et al., 2023). As part of the current investigation, virtual drug design tools were employed to screen the South African natural database against the F3 protein. The objective was to identify potent drugs capable of inhibiting the binding of the F3 protein with double-stranded RNA (dsRNA). Molecular simulation and the calculation of binding free energies were subsequently employed to validate the binding affinity between the identified top-hit compounds and the F3 protein. In essence, this study aims to offer pre-clinical proof of concept for the development of therapeutics targeting the ongoing monkeypox virus infection pandemic.

## Materials and methods

### Sequence retrieval and 3D modeling

The three-dimensional arrangement of a protein’s structure plays a crucial role in determining its functional capabilities. This intricate configuration governs how the protein interacts with other molecules and carries out specific biological tasks. To predict the 3D structure of the F3L protein, which is associated with the monkeypox virus, we retrieved its corresponding protein sequence (ID: Q5IXX3) from the UniProt database. Utilizing the AlphaFold 2.0 server, (https://colab.research.google.com/github/sokrypton/ColabFold/blob/main/AlphaFold2.ipynb#scrollTo=kOblAo-xetgx), we conducted computational modeling to anticipate the F3L protein’s three-dimensional structure. This server is capable of predicting protein structures with remarkable precision at the atomic level, even in scenarios where similar protein structures are not readily available. AlphaFold uses an attention-based neural network architecture for predicting protein structures, including a method for handling side chain conformations called “trRosetta (Jumper et al., 2021). The server generated five models and to select the best model, additional validation steps were undertaken, involving the utilization of ProSA-web analysis (https://prosa.services.came.sbg.ac.at/prosa.php) as well as Ramachandran plot (https://saves.mbi.ucla.edu/) assessment. The ProSA-web analysis serves as a crucial tool for evaluating protein structure quality, stability, and overall model validation (Wiederstein and Sippl, 2007). Concurrently, the Ramachandran plot, a visual depiction frequently employed in structural biology, was employed to scrutinize the distribution of dihedral angles within the protein’s polypeptide chain (Gopalakrishnan et al., 2007). Prior to initiating virtual drug screening, the model protein underwent a process of minimization to optimize its conformation.

### Screening of African natural drug database against the F3L protein

The 3D-SDF formatted South African natural compounds were sourced from the African Natural Products Databases (ANPDB) website (http://african-compounds.org/anpdb/), and subjected to necessary preparations for subsequent screening (Ntie-Kang et al., 2013). These databases encompass a wide array of natural products hailing from South Africa, each possessing varied medicinal attributes. Prior to subjecting these databases to computational screening procedures, the FAF-Drugs4 webserver was employed. Its purpose was to selectively retrieve compounds that are both non-toxic and exhibit drug-like characteristics, aligning with Lipinski’s rule of five (Lagorce et al., 2017). Before conducting virtual drug screening using EasyDock Vina 2.0, all drugs were converted to the.pdbqt format. For the Ligands the pdbqt format was genereated, where atomic charges and atom types were assigned using tools like Open Babel. Non-polar hydrogen atoms, Gasteiger charges, and torsion tree roots for flexibility analysis were considered for the ligand’s preparations. On the other hand, the receptor needs to be prepared as grid maps in AutoGrid to define the docking space. This involves setting the grid dimensions, spacing, and specifying the macromolecule in pdbqt format, with added hydrogen atoms, charges, and atom types. Additionally, the receptor grid was defined based on the dsRNA binding motif (80–147 amino acids). This software provides a user-friendly graphical interface for screening virtual databases. The screening process employed the AUTODOCK4 algorithm to assess and rank potential drug candidates. To facilitate quick initial screening, a lower exhaustiveness setting of 16 was selected. Subsequently, the most promising compounds, based on their scores, underwent a second screening using a higher exhaustiveness value of 64. This step aimed to eliminate false-positive outcomes and to re-evaluate the top-ranking compounds. Subsequently, out of 954 compounds the highest-ranking 10% of drugs identified through the preceding process underwent induced-fit docking (IFD) utilizing AutoDockFR. AutoDockFR typically employs force fields like AMBER or CHARMM, simulation protocols such as molecular dynamics (MD), and scoring functions like AMBER scoring or force-field-based scoring for induced-fit docking (IFD) simulations. We used the default parameters for the IFD docking. This technique accommodates receptor flexibility and facilitates a covalent docking (Ravindranath et al., 2015). The selected top four hits compounds were subjected to further processing.

### Dissociation constant (K_D_) analysis of top hits drug-F3L complex

Assessing the binding strength of a biological complex holds significance in enhancing our comprehension of specific pathways, disease mechanisms, and potential therapeutic approaches (Ma et al., 2018; Suleman et al., 2023a). To offer more accurate insights into the interactions of shortlisted drug-F3L complexes, we determined their KD values using the PRODIGY online web server (protein binding energy prediction) accessible at https://wenmr.science.uu.nl/prodigy/. This platform facilitates the computation of binding energies for both protein-protein and protein-ligand associations, while also identifying binding interfaces (Vangone et al., 2019). A higher likelihood of effectively modulating the target’s activity is indicated by a lower Kd value, which signifies a stronger binding between the drug and the protein. (Khan et al., 2022a; Suleman et al., 2022a).

### Molecular dynamic simulation of top hits-F3L complexes

We used the AMBER20 package to assess the dynamic stability of tophits-F3L complexes (Shams ul Hassan et al., 2022). The FF19SB force field and GAFF (General Amber Force Field) were used to parameterize both the protein and small drug molecules, respectively (Wang et al., 2001; Case et al., 2005). To neutralize the effect of any charge Na+ and Cl+ ions were inoculated (Price and Brooks, 2004) followed by energy minimization in two steps (algorithms: steepest descent and conjugate gradient) was achieved (Meza, 2010; Watowich et al., 1988). Following the minimization process, the system underwent equilibration and was gradually heated under a constant pressure of 1 atm at a temperature of 300 K. Subsequently, a production run of 100 ns was performed for each complex. To handle long-range electrostatic interactions with a cutoff distance of 10.0 Å, the particle mesh Ewald algorithm was employed. Meanwhile, any existing covalent bonds were managed using the SHAKE algorithm. The CPPTRAJ package was utilized for trajectory analysis, and the simulations were executed using PMEMD.cuda (Salomon-Ferrer et al., 2013; Roe and Cheatham, 2013; Khan et al., 2022b).

### Post-simulation analysis

CPPTRAJ and PTRAJ software packages were employed to assess the compactness, dynamic stability, average hydrogen bond formation, and flexibility of the tophits-F3L complexes. (Roe and Cheatham, 2013). To evaluate the structural compactness throughout the simulation period, we computed the radius of gyration (Rg) using the below mathematical formula:

$$r_{\text{RG}}^{2}=\frac{\sum_{i=1}^{N}m_{i}{\left(r_{i}-r_{\text{CM}}\right)}^{2}}{\sum_{i=1}^{N}m_{i}}$$

The assessment of structural stability was carried out by calculating the RMSD (Root Mean Square Deviation). The RMSD was calculated by using the following mathematical formula:

In order to examine the flexibility of the structure $RMSD=\sqrt{\frac{1}{N}\sum_{i=1}^{N}\delta_{i}^{2}}$ at the level of individual residues, we employed Root Mean Square Fluctuation (RMSF) computations. Throughout the simulation period, RMSF was determined by assessing the fluctuations in residues rather than the positional variations of the entire complex. The RMSF calculation was carried out by the following mathematical expression:

$$B=\frac{{8\pi }^{2}}{3}\langle {∆r}^{2}\rangle$$

By rearranging the above equation and accounting for 3 spatial dimensions, we can obtain the RMSF by the following equation:

$$RMSE=\sqrt{\frac{3B}{{8\pi }^{2}}}$$

### Binding strength analysis by calculating binding free energies

The MM/GBSA methodology was employed to compute the binding free energies of the top hits compound and F3L complexes. This technique stands out as a preferred choice in various research endeavors for accurately calculating the actual binding energies in diverse biological interactions, including protein-DNA/RNA, protein-protein, and protein-ligand interactions (Genheden and Ryde, 2015; Suleman et al., 2021). The assessment of the overall binding free energy for the top ligand complexes was performed using the MMGBSA.py script (Chen et al., 2016a). Within this analysis, each energy component, encompassing van der Waals forces, electrostatic interactions, Generalized Born solvation energy, and surface area contributions, was individually computed as integral parts contributing to the overall binding energy estimation.

The following mathematical equation was used for free energies calculation:

$$\Delta G\left(bind\right)=\Delta G\left(complex\right)\;-\;\left[\Delta G\left(receptor\right)+\Delta G\left(ligand\right)\right]$$

However, to calculate each component separately we used the following equation:

G=Gbond+Gele+GvdW+Gpol+Gnpol −TS

### Principal component analysis (PCA)

Principal Component Analysis (PCA) was employed to compute the substantial fluctuations within the protein structure (Berezin et al., 2004). Utilizing the CPPTRAJ package, the covariance matrix was derived based on the Cα coordinates, and subsequently, eigenvectors and eigenvalues were obtained through diagonalization of this matrix. PCA calculations were performed using 5,000 snapshots extracted from the trajectory of each system. The eigenvectors represent the direction of motion, while the eigenvalues indicate the magnitude of mean square fluctuation. PC1 and PC2 were utilized for visualization to track the protein’s motion. The identification of the most stable state was facilitated by examining the free energy landscape (FEL), highlighting low-energy stable states as deep valleys on the plot, while intermediate states were characterized by boundaries between these valleys (Xu et al., 2017).

### Dynamics cross-correlation analysis

The dynamics cross-correlation maps (DCCM) approach was utilized to track the sequential movements of Calcium (Ca) atoms. By employing this method, a correlation matrix was generated to comprehend the synchronized and opposing motions among C-alpha (C-a) atoms across all residues within the systems. The equation used for the DCCM calculations was:

$$Cij=<\Delta ri\;•\;\Delta rj>/\left(<\Delta ri\;2\;><\Delta rj\;2\;>\right)\;1\;/2$$

Here, the matrix Cij encodes time-correlated information between the atoms i and j. In the graphical representation, positive values denote synchronized motions, while negative values signify opposing movements observed throughout the simulation.

## Results and discussion

**FIGURE 1:**
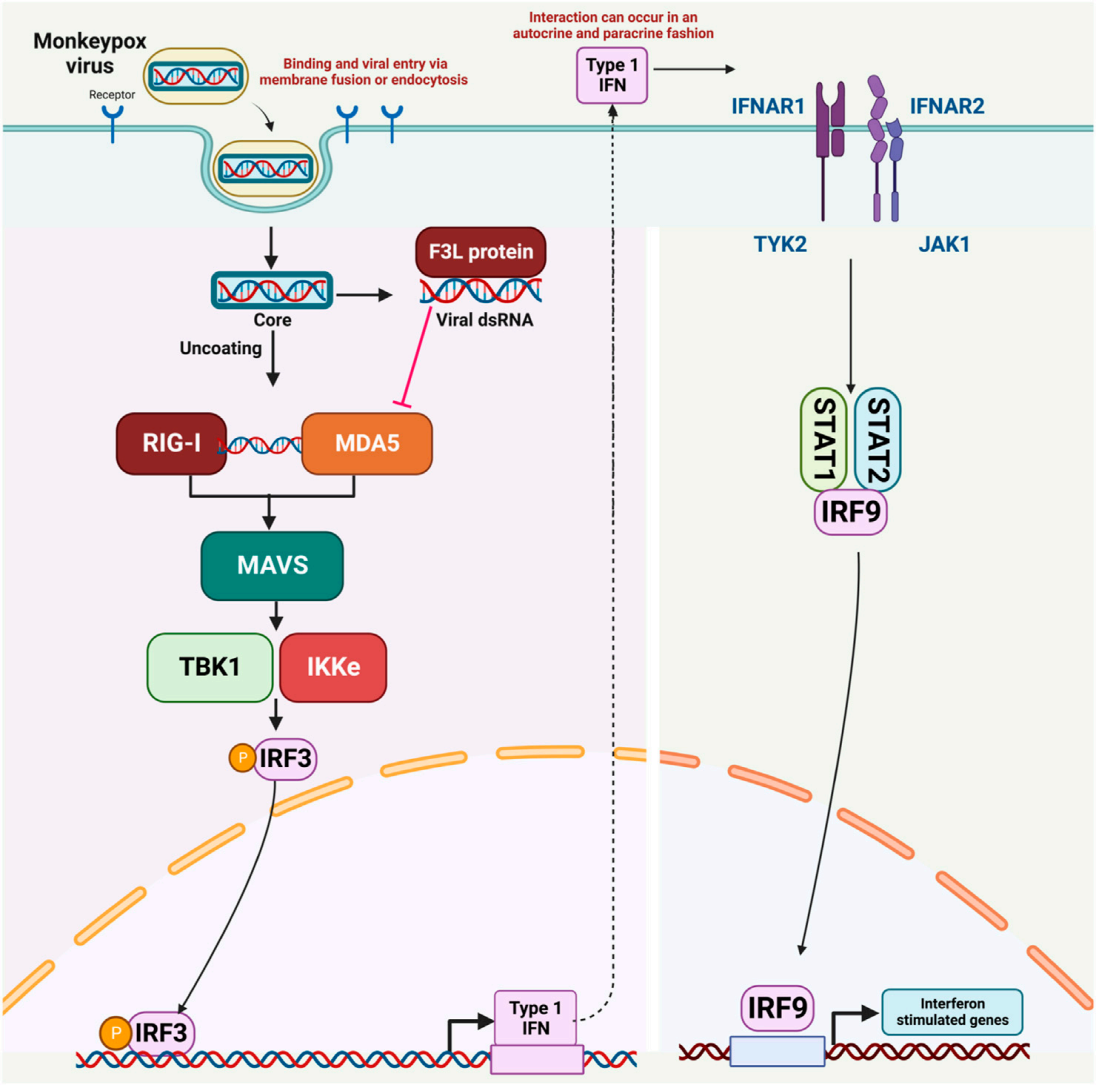
The mechanism of immune evasion by monkeypox virus.

Reports indicate that MPXV infection results in the production of double-stranded RNA (dsRNA) and the expression of the F3 protein. The F3 protein possesses the ability to bind to dsRNA molecules, effectively sequestering them from Pattern Recognition Receptors (PRRs) like MDA-5, RIG-I, and PKR. Consequently, this impedes the activation of these receptors, leading to a reduction in interferon (IFN) production through a dsRNA-dependent mechanism. (Arndt et al., 2015; Arndt et al., 2016). Given the current monkeypox pandemic, there is an urgent need for the rapid development of effective therapeutic interventions that can counteract the immune evasion mechanisms of the monkeypox virus. Consequently, our ongoing research is centered on utilizing virtual drug screening techniques to target the RNA binding domain of the F3L protein. The objective is to disrupt its interaction with double-stranded RNA (dsRNA), with the potential outcome of reducing the virus’s increased infectivity. The mechanism of immune evasion by monkeypox, as well as all molecular actors involed in this process, is shown in Figure 1.

### Structure modeling and validation

**FIGURE 2:**
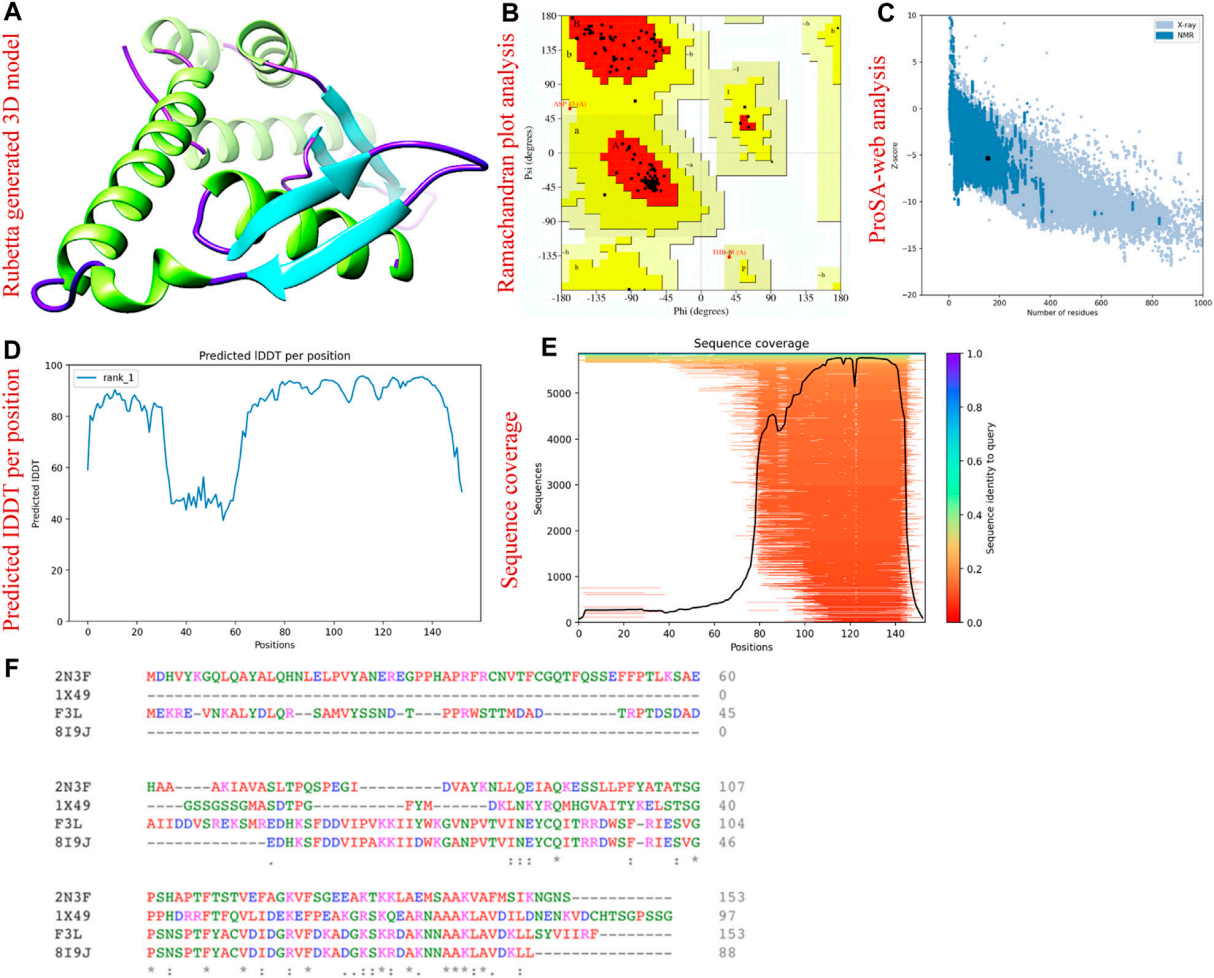
3D structural modeling and validation of F3L protein of monkeypox virus. **(A)** represents the AlphaFold generated 3D model of F3L protein. **(B)** represents the validation of F3L protein model by Ramachandran analysis. **(C)** validation of F3L protein model by ProSA-web. **(D)** represents the IDDT score per position **(E)** represents the sequence coverage of the predicted protein model. **(F)** sequence alignment of F3L and the templates used.

We used the AlfaFold 2.0 sever to predict the three-dimension structure of F3L protein of monkeypox virus. The aforementioned server is the only computational tool that can regularly predict the structure of protein with atomic level accuracy even in the cases where no similar structures are available (Jumper et al., 2021). Figure 2A is showing the AlfaFold generated 3D structure of F3L protein. To gain insight into the accuracy of the predicted structure, we used the 3D structure validation tools called the Ramachandran and ProSA-web analysis. The Ramachandran analysis revealed that the majority (95.0%) of the amino acids were found in the most favored regions while 5.0% and 0.0% amino acids were found in the additional allowed regions and disallowed region respectively (Figure 2B). Moreover, the ProSA-web assessment resulted in a Z score of −5.33 concerning the anticipated structure of the F3L protein (Figure 2C). Through a comparison of the outcomes from structural validation with data previously documented, it has been ascertained that the values fall within the typical range for protein structures of similar size (Suleman et al., 2022b). The pLDDT score was observed to be > 90 which shows the reliability of our predicted model and therefore can be used for the subsequent analysis. The sequence coverage and pLDDT graphs are given in Figures 2D, E. The alignment of the F3L protein sequence and the templates used are shown in Figure 2F.

### Virtual drug screening against the F3L protein to halt its binding with dsRNA

Virtual drug screening plays a vital role in the drug design field by expediting the identification of promising drug candidates and enhancing their chemical and biological properties (Carpenter et al., 2018; Lin et al., 2020). Natural products have shown great potential as pharmacological agents, and their discovery can be expedited through the use of advanced computational methods (Atanasov et al., 2015; Harvey et al., 2015). As part of our current investigation, we employed virtual drug screening on the South African natural product database to pinpoint lead compounds capable of targeting the F3L protein in monkeypox. The aim is to disrupt its binding with double-stranded RNA (dsRNA) and mitigate the heightened infectivity of the monkeypox virus. Prior to the database screening, we applied Lipinski’s rule of five to exclude drug-like molecules, a criterion utilized in numerous studies (Sayaf et al., 2023). The computational drug screening was performed using AutoDock Vina, targeting the F3L protein of the monkeypox virus. From a pool of 954 compounds in the South African database, we retained 823 molecules based on Rule of Five criteria. These 823 compounds underwent a multi-step screening approach. Initially, the first round of virtual screening produced docking scores spanning from −6.38 to 3.82 kcal/mol. After this round, we selected compounds with docking scores ranging from −6.38 to −5.00 kcal/mol. These selected compounds were then subjected to induced-fit docking. In this phase, induced-fit docking yielded docking scores ranging from −6.55 to −4.50 kcal/mol. Among the screened compounds, only 17 showed docking scores higher than −6.0 kcal/mol and demonstrated favorable interaction profiles. Based on the high docking score only four compounds, namely, (3R,5S)-3-[(3S,5S,9R,10S,13S,14R,16R,17R)-16-hydroxy-10,13-dimethyl-3-[(2R,3S,4S,5R,6S)-3,4,5-trihyd, [(2S, 3S,6E,10E,14E,18R)-2,3,18,19-tetrahydroxy-2,6,11,15,19-pentamethyl-icosa-6,10,14-trienyl], 8-oxo-16-[(2R,3S,4S,5S,6R)-3,4,5-trihydroxy-6-(hydroxymethyl)tetrahydropyran-2-yl]oxy-hexadecanoic, (2R)-2-[(3S, 4S)-3,4-dihydroxy-2,2-dimethyl-8-(4-methylpent-3 -enoxy)chroman-6- yl]-5,7-dihydroxy-chrom with a docking score of −6.55 kcal/mol, −6.47 kcal/mol, −6.37 kcal/mol and −6.35 kcal/mol respectively. A similar approach has been used by the previous study which uses the FDA approved drugs against the three different targets such as topoisomerase1, p37, and thymidylate kinase of the monkey pox virus (Srivastava et al., 2023). Another study used drug screening against monkey pox virus cysteine proteinase (Odhar, 2022). The drugs IDs, 2D structures, name and docking score are shown in Table 1.

**TABLE 1:** List of top hits compounds along with structures and docking scores.

Drug IDs	2D structure	Name	Docking score
EA_0096	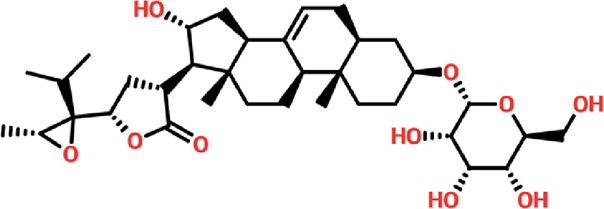	(3R,5S)-3-[(3S,5S,9R,10S,13S,14R,16R,17R)-16-hydroxy-10,13-dimethyl-3-[(2R,3S,4S,5R,6S)-3,4,5-trihyd	−6.55 kcal/mol
EA_0082	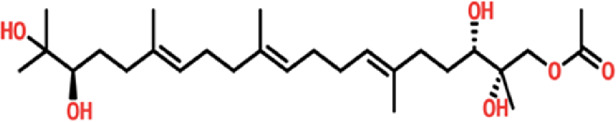	[(2S,3S,6E,10E,14E,18R)-2,3,18,19-tetrahydroxy-2,6,11,15,19-pentamethyl-icosa-6,10,14-trienyl]	−6.47 kcal/mol
SA_0090	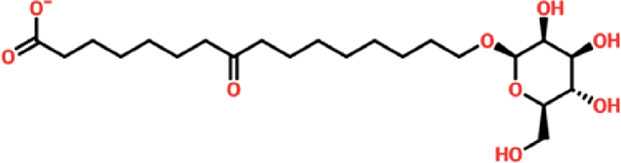	8-oxo-16-[(2R,3S,4S,5S,6R)-3,4,5-trihydroxy-6-(hydroxymethyl)tetrahydropyran-2-yl]oxy-hexadecanoic	−6.37 kcal/mol
BMC_00079	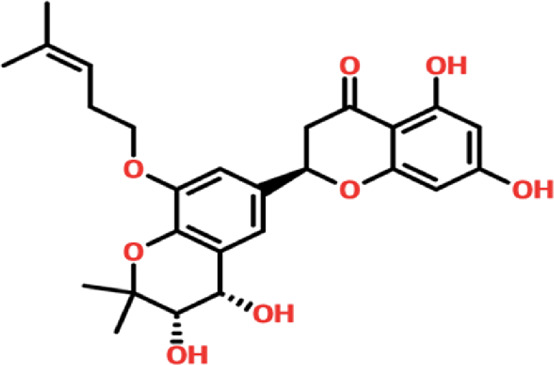	(2R)-2-[(3S,4S)-3,4-dihydroxy-2,2-dimethyl-8-(4-methylpent-3-enoxy)chroman-6-yl]-5,7-dihydroxy-chrom	−6.35 kcal/mol

### Bonding analysis of tophits-F3L protein complexes

**FIGURE 3:**
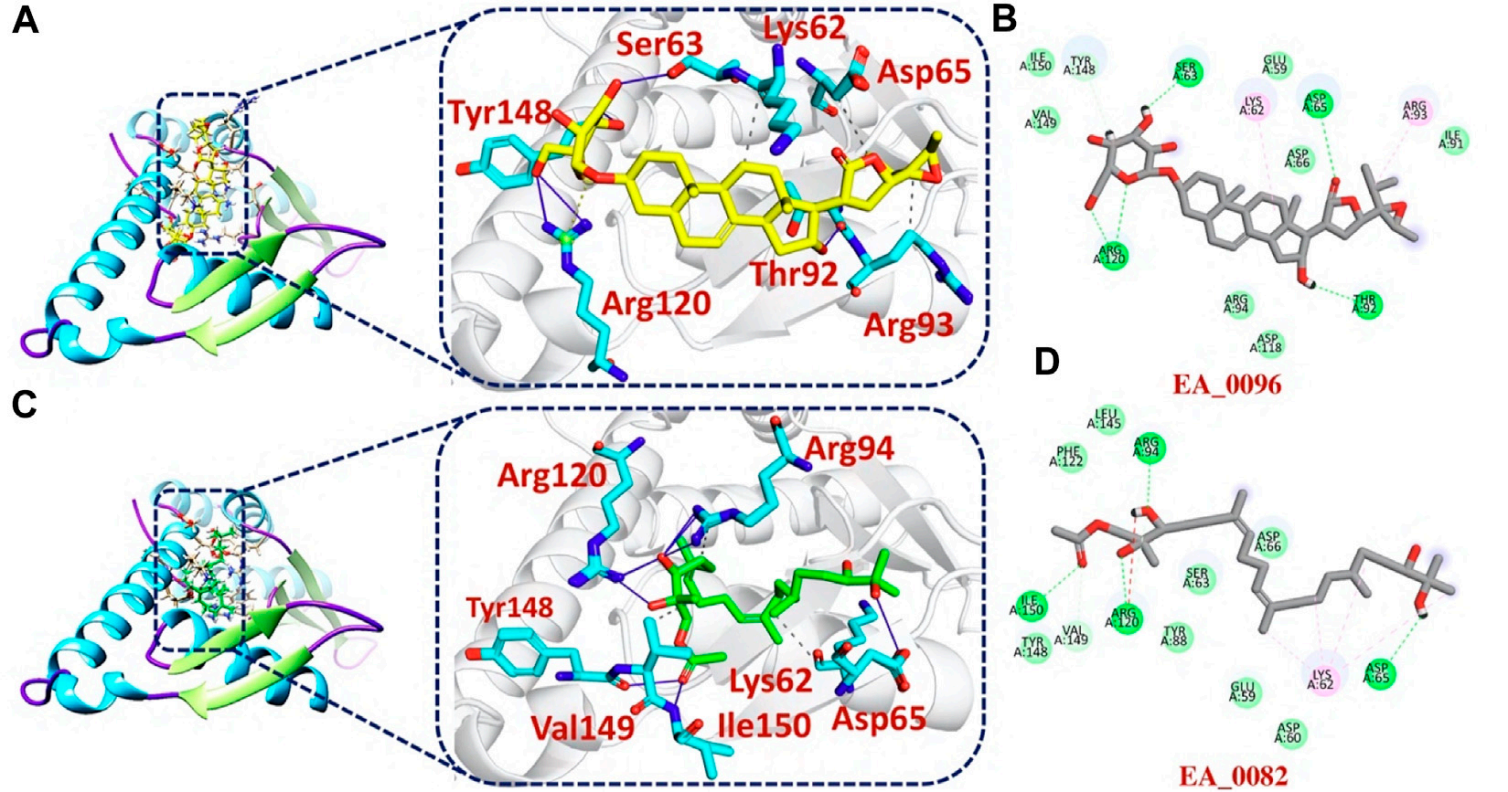
2D and 3D interaction of top hit1 and 2 with F3L protein. **(A)** showing the bonding network between tophit1 and F3L protein as a 3D interaction **(B)** 2D interaction between top hit 1 and F3L protein **(C)** showing the bonding network between tophit2 and F3L protein as a 3D interaction **(D)** 2D interaction between top hit 2 and F3L protein.

The bonding network analysis of tophits-F3L protein complexes revealed the formation of hydrogen bonds, salt bridges and hydrophobic interaction between the drug and target protein. The examination of the tophit1-F3L complex unveiled a docking score of −6.55 kcal/mol, showcasing the presence of one salt bridge, seven hydrogen bonds, and three hydrophobic interactions. The bonding network between the top hit1 compound and the target protein was primarily mediated by specific amino acids, namely, Lys62, Ser63, Asp65, Thr92, Arg93, Arg120, and Tyr148, as depicted in Figures 3A, B. Similarly, our analysis of the top hit2-F3L complex revealed the formation of 11 hydrogen and 3 hydrophobic interaction with a docking score of −6.47 kcal/mol. The key hotspot residues Lys62, Asp65, Arg94, Arg120, Tyr148, Val149 and Ile150 were involved in hydrogen and hydrophobic bonds formation (Figures 3C, D).

**FIGURE 4:**
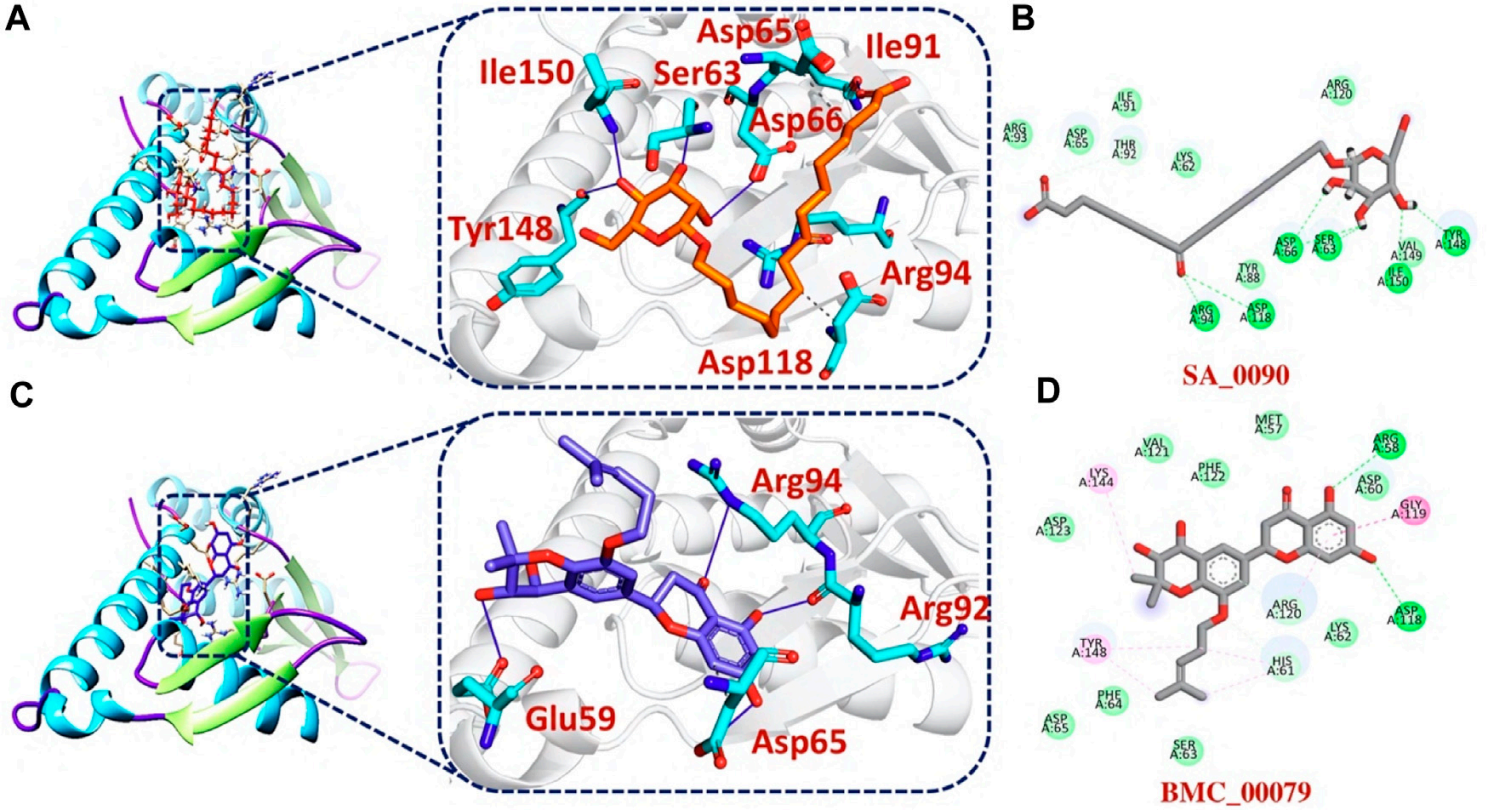
2D and 3D interaction of top hit3 and 4 with F3L protein **(A)** showing the bonding network between tophit3 and F3L protein as a 3D interaction **(B)** 2D interaction between top hit 3 and F3L protein **(C)** showing the bonding network between tophit4 and F3L protein as a 3D interaction **(D)** 2D interaction between top hit 4 and F3L protein.

Additionally, our analysis of the bonding network indicated that the top hit 3 compounds displayed highly favorable interactions with the F3L protein. Specifically, it formed a total of 9 hydrogen bonds and 3 hydrophobic bonds with particular amino acids. These amino acids include Ser63, Asp65, Asp66, Ile91, Arg94, Asp118, Tyr148, and Ile150, as illustrated in Figures 4A, B. Finally, the interaction analysis of top hit 4-F3L complex found the existence of 4 hydrogen bonds and 2 hydrophobic interactions with the docking score of −6.35 kcal/mol. The key residues involved in the bonding network of the top hit4-F3L complex were Glu59, Asp65, Thr92, and Arg94 (Figures 4C, D). Our results indicate that these compounds hold substantial promise as potential drug candidates. They display favorable interactions with the target protein, effectively hindering the binding of the F3L protein to dsRNA, which could potentially diminish the monkeypox virus ability to evade the immune system.

### Dissociation constant (KD) analysis of top hits-F3L complexes

We calculated the KD values for the complexes of all the top-hit compounds with F3L using the PRODIGY approach. PRODIGY, short for PROtein binDIng enerGY, operates as a web server specifically designed for predicting binding affinity in both protein-protein and protein-ligand complexes. Leveraging fundamental structural features, such as interactions between residues at the interface, PRODIGY has demonstrated remarkable predictive capabilities, surpassing existing state-of-the-art predictors in the field. Moreover, the PRODIGY-LIG extension, tailored for small ligands, has been improved to utilize atomic contacts at the interface for more accurate predictions. (Vangone et al., 2019). PRODIGY analysis revealed the binding scores of −5.34 kcal/mol for top hit1-F3L complex, −5.32 kcal/mol for top hit2-F3L complex, −5.29 kcal/mol for top hit3-F3L complex, and −5.36 kcal/mol for top hit4-F3L complex. These scores indicated the strong binding affinity of these compounds with the F3L protein target. Our findings coincide with various comparable studies that have also reported similar range of scores indicating a heightened binding affinity within the protein-ligand complexes (Khan et al., 2023; Suleman et al., 2023b). This heightened affinity suggests the potential of these molecules to effectively hinder the F3L protein’s interaction with dsRNA during monkeypox virus infection.

### Dynamic stability analysis of top hits-protein complexes by MD simulation

**FIGURE 5:**
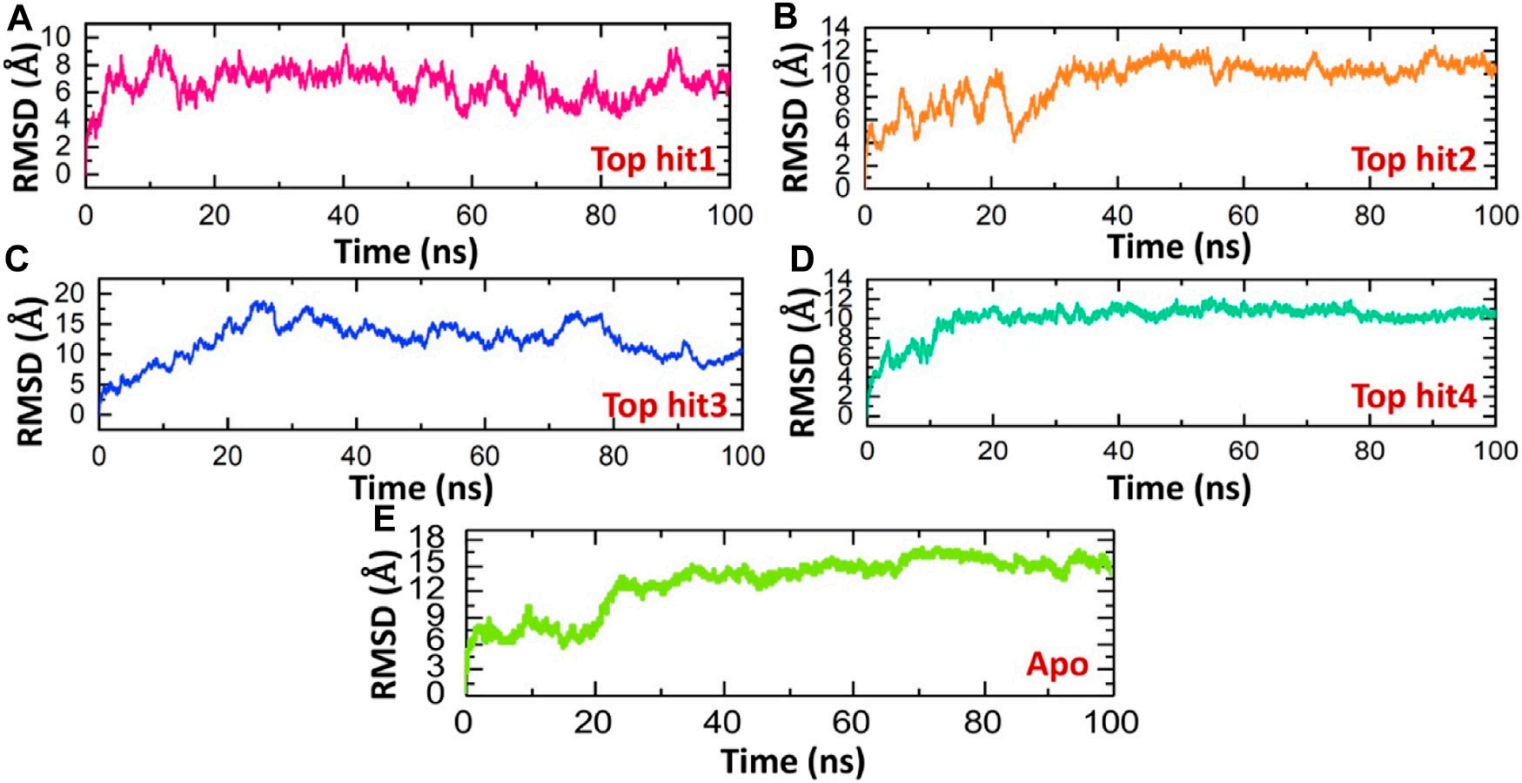
Dynamic stability analysis of top hits and protein as RMSD. **(A)** showing dynamic stability of tophit1-protien complex as RMSD value **(B)** showing dynamic stability of tophit2-protien complex as RMSD value **(C)** showing dynamic stability of tophit3-protien complex as RMSD value **(D)** showing dynamic stability of tophit4-protien complex as RMSD value **(E)** showing dynamic stability of apo protein as RMSD value.

Evaluating the dynamic stability of a protein when it's bound to a ligand is crucial for establishing the pharmacological efficacy of that specific compound (Liu et al., 2017; Jalal et al., 2022). To ascertain the stability of these simulations, we employ the Root Mean Square Deviation (RMSD) function integrated into simulation tools. Therefore, in order to assess whether these compounds maintain their stability throughout the simulation, we have computed the RMSD values for these trajectories over time. The RMSD of the tophit1-protien complex stabilized at 6.0 A and remained stable throughout the time of simulation however, only minor deviations were observed at different time points. The average Root Mean Square Deviation (RMSD) for the first complex was measured at 6.0 Å, displaying a consistently straight line pattern, indicative of the stable dynamics maintained throughout the simulation period (Figure 5A). Conversely, the tophit2-protein complex exhibited a more variable behavior, with an average RMSD of 10 Å. However, a minor fluctuation was noted between 10 and 20 ns, introducing some variability into the stability profile of this complex (Figure 5B). In the case of the tophit3-protein complex, the RMSD initially increased up to the 30 ns mark, followed by a gradual decrease for the remainder of the simulation, without significant deviations. The average RMSD for the tophit3-protein complex was approximately 10 Å (Figure 5C). Finally, the tophit4-protein complex achieved equilibrium at 10 ns with an RMSD value of 10 Å, maintaining stability until the end of the simulation. The average RMSD for this complex showed a consistent straight line throughout the simulation time period (Figure 5D).Similar stability results were found previously for the analysis of drugs against the different targets such as thymidylate kinase (Khan et al., 2023), E8 surface protein (Podduturi et al., 2023), of monkey pox virus. In comparison, the tophit1-protein complex demonstrated superior stability, evidenced by its lower RMSD value. The evaluation of the dynamic stability of these complexes suggests that they present a consistent pharmacological profile, possibly contributing to improved pharmacological efficacy in an *in vitro* experimental context. Figure 5 displays the Root Mean Square Deviation (RMSD) graphs for the top four hits. It can be seen that the apo state determined the higher RMSD levels and therefor show that these ligands upon the binding produces structural constraints that consequently stabilizes the structure and reported minimal RMSD in the complex states (Figure 5E).

### Structural compactness analysis as Rg for the top four hits complexes

**FIGURE 6:**
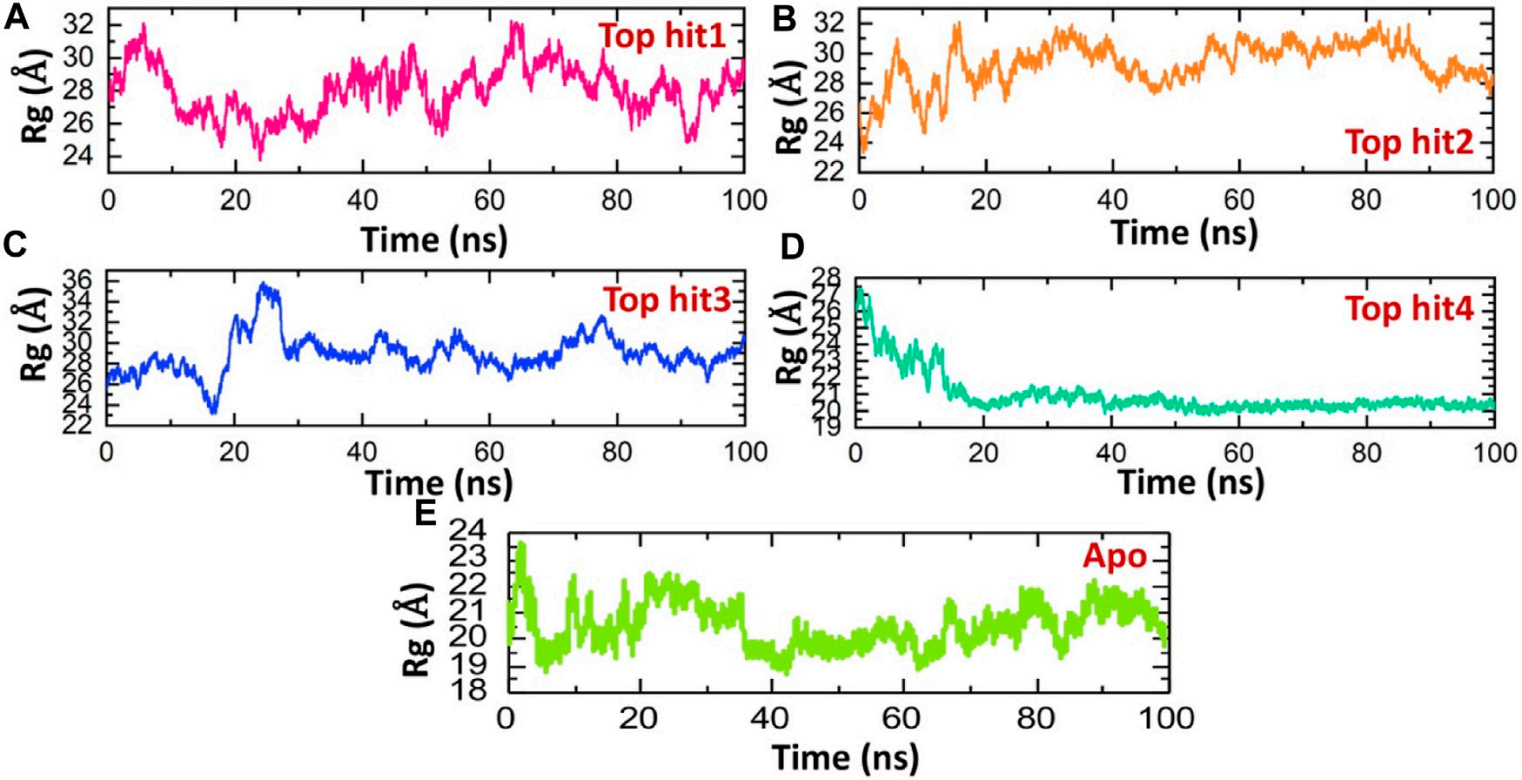
Compactness analysis of top hits-protein complexes and apo protein as Rg. **(A)** showing the Rg value of tophit1-protien complex **(B)** showing the Rg value of tophit2-protien complex **(C)** showing the Rg value of tophit3-protien complex **(D)** showing the Rg value of tophit4-protien complex **(E)** showing the Rg value of protein alone.

This methodology has previously proven effective in elucidating how interactions between different proteins or protein-ligand complexes influence a protein’s behavior over time (Khan et al., 2022c; Khan et al., 2022d). Here, we employed this approach to assess the structural compactness of each complex throughout the simulation, achieved by tracking the radius of gyration (Rg) over time. Notably, the observed Rg patterns for the top complexes closely mirror the findings obtained from RMSD analysis. The Rg (Radius of Gyration) value for the top hit1-protein complex initially decreased to approximately 24 Å, followed by a subsequent increase to 30 Å.This brief increase in Rg was later followed by a return to its lower value. On average, the Rg value for the top hit1-protein complex was measured at 28 Å as shown in Figure 6A. These fluctuations in Rg values indicate the binding and unbinding events of the ligand within the binding pocket. Conversely, in the case of the top hit2-protein complex, the system reached equilibrium at 20 ns with a stable Rg value of 28 Å, which remained consistent throughout the simulation. The average Rg value for the top hit2-protein complex was also recorded as 28 Å as depicted in Figure 6B. Similarly, in case of tophit3-protien complex the Rg value was significantly perturbated between 10 and 30ns and then remined stable until the end of simulation. The average Rg value for the tophit3-protien complex was found to be 27 A (Figure 6C). Finally, the tophit4-protien complex was found to be the most compact as compared the other three complexes with the average Rg value of 20 A (Figure 6D). This complex was equilibrated at 20ns and remained stable till the end of simulation. A similar approach has been used by previous studies to check the compactness of the drugs and different monkey pox protein targets (Dutt et al., 2023). These findings suggest that the tophits-protein complexes possess a compact structure, potentially leading to potent pharmacological effects against the protein. Similarly, the app simulation Rg shows a compact nature and therefore determined a similar result as the RMSD where the structure reported stable dynamics behavior (Figure 6E).

### Fluctuation analysis of tophits-F3L complexes at residues level

**FIGURE 7:**
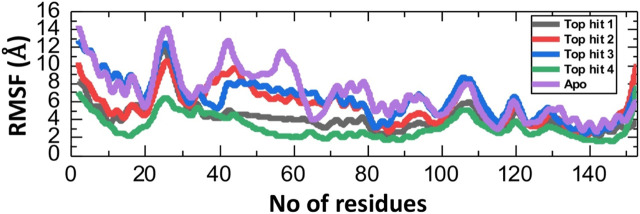
Residual fluctuation analysis of top hits-protein complexes and apo protein as RMSF.

The Root Mean Square Fluctuation (RMSF) calculation is a valuable tool in the study of protein-drug interaction in molecular biology and structural bioinformatics (Fatriansyah et al., 2022; Ghufran et al., 2022). It provides critical insights into the dynamic behavior of proteins and can be particular important in the understanding how proteins interact with drugs (Rashid et al., 2021; Shah et al., 2022). To check the fluctuation of top hit-protein complexes at residues level we calculated the RMSF value during the 100ns simulation. As depicted in Figure 7, the tophit4-protein complex demonstrates a notable equilibrium state among its residues, with an average RMSF value of 4 Å. Conversely, a similar RMSF pattern was observed for the tophit1-3-protein complexes, displaying significant fluctuations within the range of amino acid residues 20-30. Additionally, fluctuations were observed in the 100-110 region. Higher RMSF values at specific residues indicate increased flexibility in those regions, while lower values signify minimal deviation from their average positions during the simulation. Ultimately, these variations in dynamic flexibility lead to diverse conformational optimization and binding interactions with the protein. Interestingly the apo reported higher fluctuation which shows that in the ligand bound complexes the internal fluctuation is stabilized by these ligands and thus produces robust pharmacological properties in contrast to the apo state.

### The average hydrogen bonding analysis of top hits-protein complexes

**FIGURE 8:**
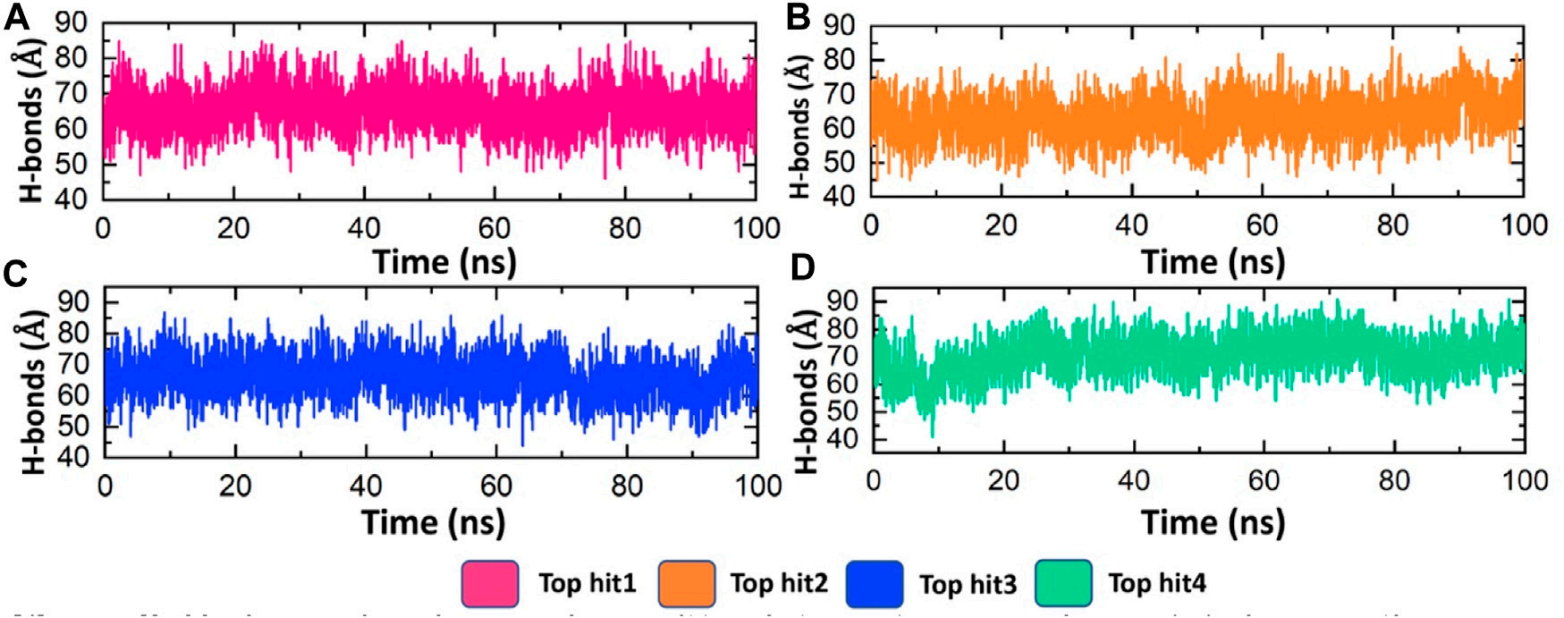
Hydrogen bonding analysis of top hits-protein complexes. **(A)** showing the average hydrogen bonds in the tophit1-protien complex **(B)** showing the average hydrogen bonds in the tophit2-protien complex **(C)** showing the average hydrogen bonds in the tophit3-protien complex **(D)** showing the average hydrogen bonds in the tophit4-protien complex.

Hydrogen bonding plays a pivotal role in biological systems by facilitating robust intermolecular connections, thereby promoting molecular recognition and enabling essential biological processes (Chen et al., 2016b). Hydrogen bond analysis was conducted on all complexes to determine the number of hydrogen bonds formed in each frame of the molecular dynamics (MD) simulation. As depicted in Figure 7, the targeted complexes exhibit a robust network of hydrogen bonds, underscoring the strong stability of the bound compounds with the protein. Specifically, the average number of hydrogen bonds observed for tophit1-4 were 70, 65, 70, and 75, respectively (Figures 8A–D). This substantial stability, as evidenced by the RMSD and RMSF data discussed earlier, can be attributed to the extensive hydrogen bonding within the systems. This could potentially lead to the inhibition of the target protein, thereby reducing the infectivity of the monkeypox virus.

### Binding free energies calculation for tophits-F3L complexes

Binding free energy calculation is commonly adopted strategy for investigating the strength of molecular interactions and uncovering the pivotal binding characteristics that govern the overall binding process (Wang et al., 2019; Khan et al., 2021). We employed the MM/GBSA approach to compute the binding free energy of complexes involving the top hits with F3L protein of monkeypox virus. The calculated values for the Van der Waals energies of top hit 1-4 complexes showed distinct stability of intermolecular forces. The recorded values were recorded to be −48.2627 kcal/mol, −43.2109 kcal/mol, −34.2082 kcal/mol, and −42.3340 kcal/mol for the respective complexes. These values underscore the attractive forces between molecules due to Van der Waals interactions, offering insights into the stability and affinity of these complexes. Furthermore, the electrostatic energies calculated for the same complexes offer a glimpse into their charged interactions. The computed electrostatic energies are found to be −15.0011 kcal/mol, −195.2000 kcal/mol, −70.9841 kcal/mol, and −5.8987 kcal/mol, revealing the extent to which electrostatic forces contribute to the overall binding energy. The binding free energies for the ESURF, EGB, Delta G Gas, and Delta G Solv were also calculated as shown in Table 2. These values shed light on the thermodynamic stability of the complexes in various environments. Notably, the total binding free energy results are depicted as −35.9079 kcal/mol, −52.7409 kcal/mol, −28.1702 kcal/mol, and −32.1119 kcal/mol for the top hit1-4 complexes. Previously, the natural products were screened against the thymidylate kinase from Monkey pox virus and have found the similar range of binding free energies and concluded the high binding affinity of the ligand protein complexes (Khan et al., 2023). Furthermore, we also calculated the MM/PBSA for the last 20ns of the simulation, In case of MM/PBSA the total binding free energies for the top hit1-4 was recorded to be −30.1667 kcal/mol, −47.3486 kcal/mol, −21.2884 and −24.8321 kcal/mol respectively. These values encapsulate the overall energetic favorability of the complex formation process, with higher negative values indicating stronger binding and more stable interactions between the molecules. The results suggest that the compounds (top hit1-4), exhibit notable binding affinity with the target F3L protein of the monkeypox virus. This binding affinity could potentially hinder the interaction between F3L and dsRNA, thereby potentially limiting the monkeypox virus’s capacity to evade the human immune system. Given these outcomes, it is highly advisable to prioritize the top hit drugs for further investigation through both *in vivo* and *in vitro* experiments.

**TABLE 2:** Binding free energies calculation by MM/GBSA approach.

MM/GBSA				
Parameters	Top hit 1-F3L	Top hit 2-F3L	Top hit 3-F3L	Top hit 4-F3L
ΔEvdw	−48.2627	−43.2109	−34.2082	−42.3340
ΔEele	−15.0011	−195.2000	−70.9841	−5.8987
ESURF	−5.1196	−6.9751	−5.1203	−4.9830
EGB	32.4755	192.6451	82.1424	21.1039
Delta G Gas	−63.2638	−238.4109	−105.1923	−48.2328
Delta G Solv	27.3559	185.6700	77.0221	16.1209
∆G total	−35.9079	−52.7409	−28.1702	−32.1119

MM/PBSA				
Parameters	Top hit 1-F3L	Top hit 2-F3L	Top hit 3-F3L	Top hit 4-F3L
vdW	−48.2627	−43.2109	−34.2082	−42.334
EEL	−15.0011	−195.2000	−70.9841	−5.8987
EPB	46.5471	205.2701	105.1224	37.5169
ENPOLAR	−13.45	−14.2078	−21.2185	−14.1163
DELTA G gas	−63.4110	−178.2057	−52.1359	−45.3581
DELTA G solv	31.0812	69.3008	53.1398	44.3410
DELTA TOTAL	−30.1667	−47.3486	−21.2884	−24.8321

### PCA and free energy landscape analysis

**FIGURE 9:**
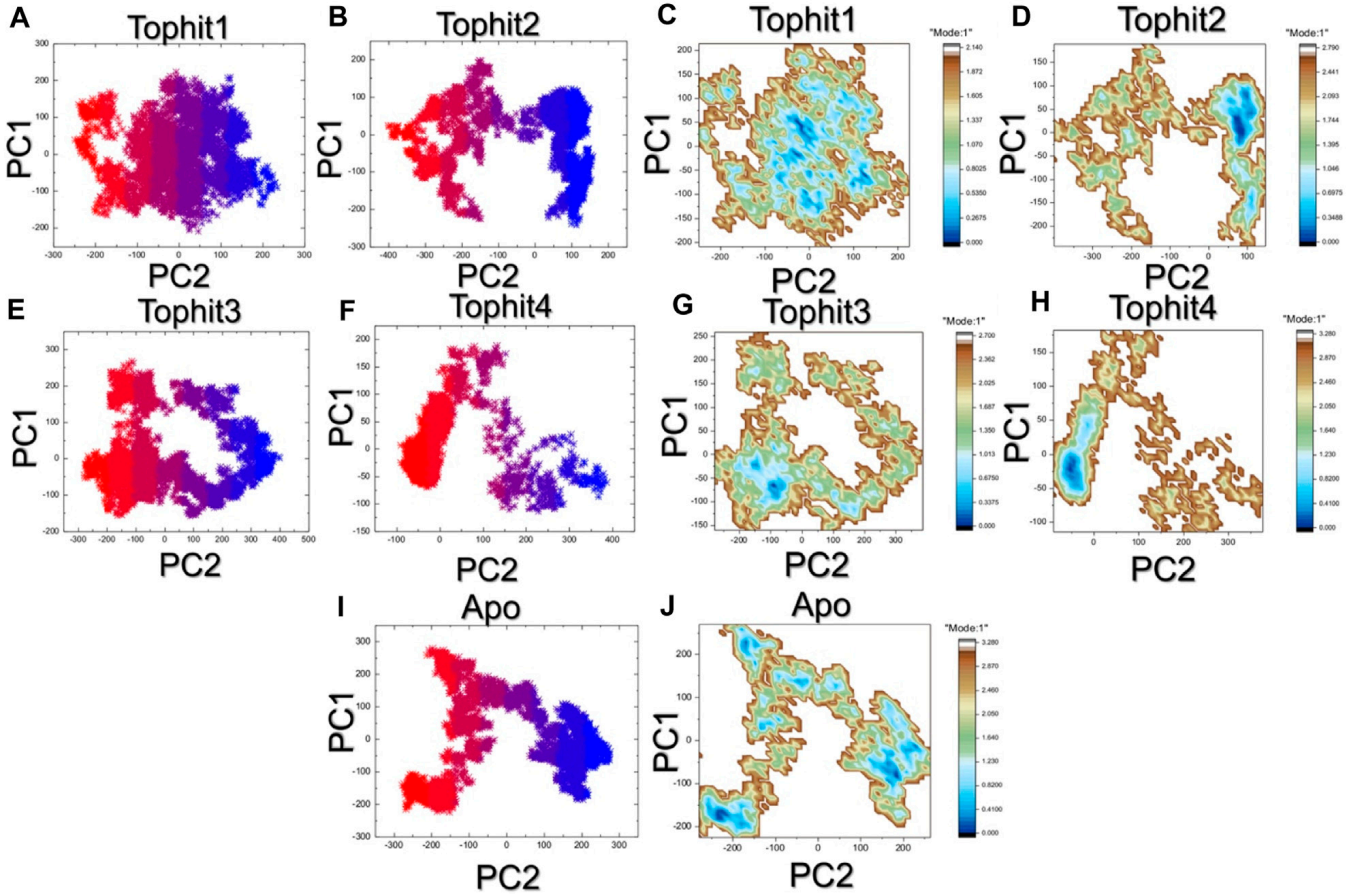
The PCA and free energy landscape analysis of top hits. **(A–E)** Principle component analysis of the top hits complexes and apo protein. **(F–J)** free energy landscape of the top hits complexes and apo protein.

The principal component analysis (PCA) approach was used to cluster the simulation trajectories which revealed different dynamic behavior presented by each complex. for instance, the top hit 1 and top hit 3 reported alike behavior which was observed to be more wide spread than the top hit2 and top hit 4 that revealed conformational transition in wide spread *x* and *y*-axis. This pattern can be separated from each other by observing the conformational transition (purple color) and each conformation which was obtained can be seen as blue and red color. The PCA results for each complex are given in Figures 9A–E. Then the two PCs were mapped onto the *x* and *y*-axis plan and the lowest energy states were observed. The top hit 1 reported multiple lowest energy conformations while the other complexes reported only single lowest energy conformation. This shows that the differential pharmacological effects produced by these ligands upon the binding. A non-constraint motion in the apo state was observed which further determined the variations in the internal motion and FEL in contrast to the holo state. The FEL results are given in Figures 9F–J.

### Contact map analysis of top hits and F3L protein

**FIGURE 10:**
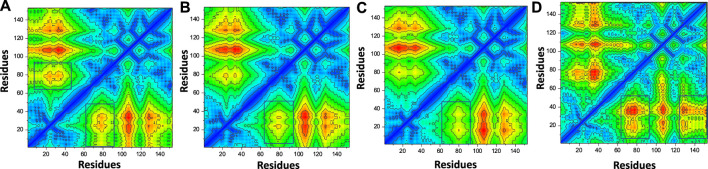
Contact map analysis of the top hits-F3L protein complexes. **(A)** showing the contact map analysis of top hit 1-F3L complex. **(B)** showing the contact map analysis of top hit 2-F3L complex. **(C)** showing the contact map analysis of top hit 3-F3L complex. **(D)** showing the contact map analysis of top hit 4-F3L complex.

In order to estimate the conformational dynamics variations, we calculated dynamic-cross correlation map (DCCM) using the simulation trajectory. For instance, the region 60-80 region demonstrated difference in the conformational dynamics and therefore shows the variations in the internal dynamics and fluctuations during the simulation. Furthermore, the top hit 4 regions also reported variations in the region 130-160 and therefore reported dynamically different correlation. The overall results show that the binding of these ligands particularly affect 60-80 region and therefore produce dynamically variable results. The contact map results are given in Figures 10A–D.

## Conclusion

In response to the ongoing monkeypox outbreak, there is an imperative need to swiftly develop therapeutic solutions that can effectively counteract the immune evasion mechanisms employed by the monkeypox virus (MPXV). A key strategy utilized by MPXV involves the F3L protein binding to double-stranded RNA (dsRNA), leading to a reduction in interferon (IFN) production. To address this, we applied structure-based drug design, conducted binding free energy calculations, and employed molecular simulation techniques to disrupt the interaction between the F3L protein and dsRNA. Through screening the African natural compound database, we identified four compounds with docking scores of −6.55 kcal/mol, −6.47 kcal/mol, −6.37 kcal/mol, and −6.35 kcal/mol. Further validation through dissociation constant analysis, molecular dynamics simulations, and binding free energy calculations supported the pharmacological efficacy of these compounds against the F3L protein. In conclusion, this study provides a compelling rationale for the development of therapeutics targeting the immune evasion mechanism of the monkeypox virus.

## Data Availability

The original contributions presented in the study are included in the article/Supplementary Material, further inquiries can be directed to the corresponding authors.
